# Employment history indicators and mortality in a nested case-control study from the Spanish WORKing life social security (WORKss) cohort

**DOI:** 10.1371/journal.pone.0178486

**Published:** 2017-06-01

**Authors:** María Andrée López Gómez, Laura Serra, George L. Delclos, Fernando G. Benavides

**Affiliations:** 1 Center for Research in Occupational Health (CiSAL), Department of Experimental and Health Sciences, Pompeu Fabra University, Barcelona, Spain; 2 CIBER of Epidemiology and Public Health, Madrid, Spain; 3 IMIM Parc Salut Mar, Social epidemiology and occupational health group, Barcelona, Spain; 4 Department of Epidemiology, Human Genetics and Environmental Sciences, The University of Texas School of Public Health (UT), Houston, TX, United States of America; Ruhr-Universitat Bochum, GERMANY

## Abstract

Employment has transitioned from stable to more flexible schemes. Little is known about the effects of dynamic working lives and mortality. We examined the association of employment, unemployment and inactivity on mortality among workers born in 1926–1988, in a nested case-control study of workers from the Spanish WORKss cohort. Cases were all deaths that occurred during 2004–2013 and controls were living persons, matched for sex and age at the time the case occurred. We had information on employment from 1981 to 2013. Logistic regression was used to measure the associations between the 3 employment history indicators separately by sex. There were 53,989 cases and an equal number of controls (n = 107,978). More than 16 years employed showed a protective effect against mortality in women (OR = 0.88, 95%CI: 0.81, 0.94) and men (OR = 0.76, 95%CI: 0.70, 0.79). The number of spells and time in unemployment and inactivity were significantly related to mortality in men, but not women. Sensitivity analyses by labor relationship showed stronger associations of unemployment (OR = 1.42, 95%CI: 1.13, 1.78) and inactivity (OR = 1.34; 95%CI: 1.09, 1.65) for temporary workers. Employment gaps are detrimental to health and have worse effects if the gaps occur without unemployment benefits or after temporary contracts. These results may drive improvement of labor and social policies that protect workers against the potential negative effects of dynamic work lives.

## Introduction

Employment, a key determinant of health, has been deeply affected by changes in the global economic market and worldwide crisis. In recent decades, these changes have transformed employment patterns from stable and predictable to more flexible and, at times, uncertain [1]. These new employment arrangements include temporary contracts and part-time jobs, coupled with less rigid labor policies intended to promote greater production. As a consequence, the probability of leaving and entering the labor market has increased [2], and it is well known that unemployment is a risk factor for both morbidity and mortality [3–8]. This association persists even in countries with generous welfare systems [9] that are designed to cushion the negative effects of unemployment on health by providing equal standards of social protection [10–13]. In this sense, new employment trends are being coupled with welfare state structures known to impact health [10] through mechanisms of welfare redistribution that depend on several factors: eligibility criteria, duration and generosity of benefits [12], and policies targeting family-work balance [14] and integration in the labor market [15].

To better understand the impact of labor market changes, both positive and negative, on health and how the welfare state is adapting to them, we can examine the relationships between employment patterns, social benefits and health. One approach is to study employment history, which covers periods of employment, unemployment and inactivity. To date, most studies have focused on baseline measurements of employment [6, 16–17], while assessing morbidity or mortality at later stages. However, current employment histories are increasingly more dynamic, and cumulative time spent in both stable and precarious employment states may well be an important determinant of health, including mortality. Given previous evidence of the role that unemployment and job insecurity play in health, our hypothesis was that unstable employment history characterized by several contracts, various periods of unemployment and employment inactivity and more time in either of the latter two states is detrimental to health. With this in mind, we examined the effect of three indicators of employment history, employment, unemployment and inactivity, on mortality among workers registered in the Spanish social security system.

## Methods

### Study design and sample selection

**Fig 1:**
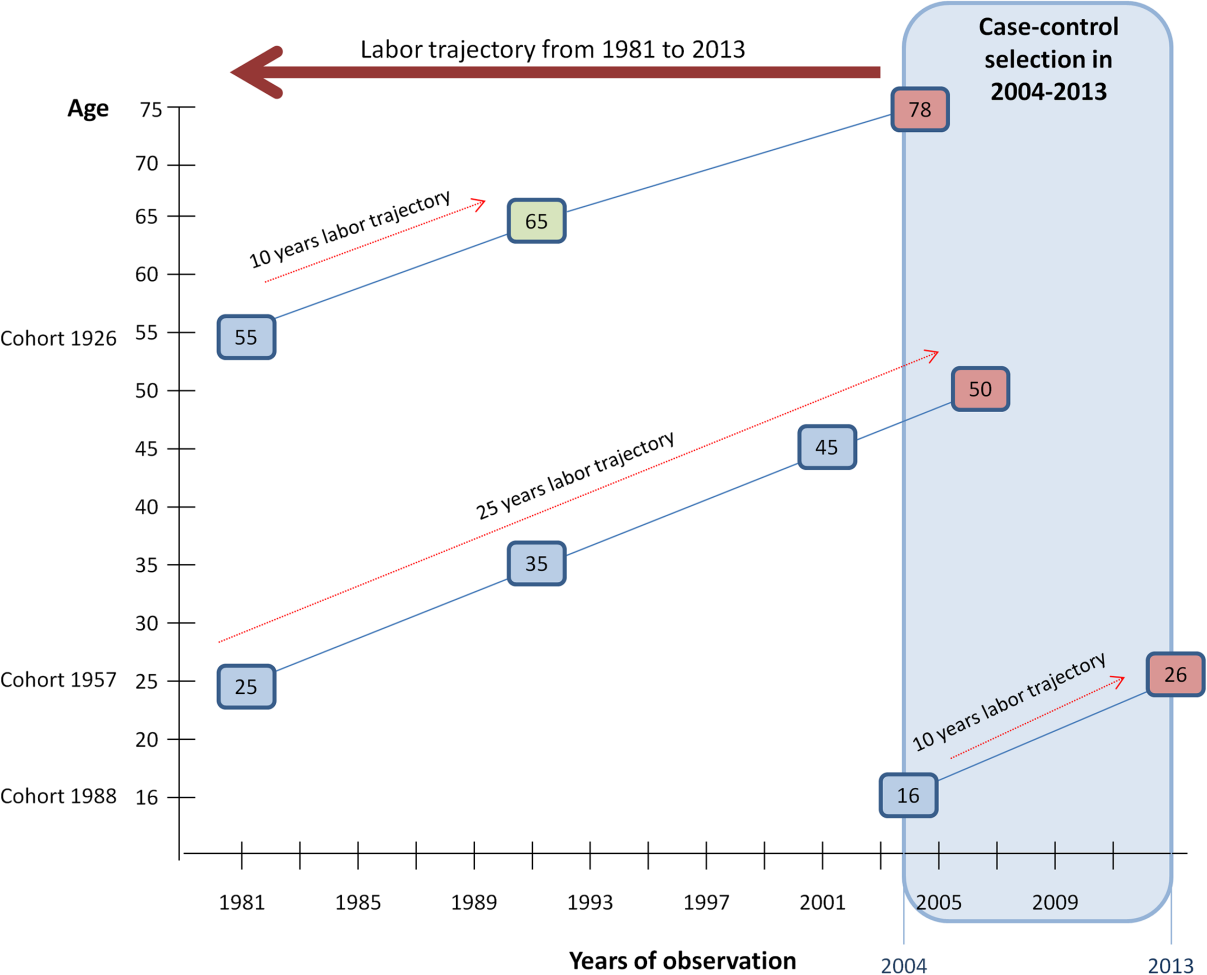
Follow-up time of employment history in three different birth cohorts (1926, 1957, 1988) of the nested case-control sample from the Spanish WORKing Life Social Security (WORKss) cohort study 2004–2013.

This was a nested case-control study conducted in a sample of workers from the Spanish WORKing life Social Security (WORKss) cohort. WORKss is a longitudinal study based on a 4% annual random sample of members of Spain´s social security system, from 2004 to 2013 [18]. Cases and controls were selected from among individuals with at least one registered job contract over the observation period. Cases were defined as individuals who died during the period 2004 to 2013. The WORKss cohort study registers the date of death of individuals who remain in contact with the social security system. Controls were matched for age and sex to the case, but were alive at the time of random selection. The rationale behind the study design is that cases and controls will have been exposed to the same labor market changes throughout their life at the same ages. Since the cohort contains administrative records dating back to 1981, we limited the sample to individuals born during the period 1926–1988 (Fig 1) in order to obtain at least a 10-year window of employment history, while also considering that the legal ages for starting work and retirement in Spain are 16 and 65 years, respectively. Thus, persons born in 1926 would have been age 55 in 1981, allowing us to obtain at least 10 years of working life follow-up before they retired at age 65. Likewise, those born in 1988 were 16 years old in 2004, also allowing a follow-up window of 10 years between 2004 and 2013. The employment history of the controls was truncated at the time of their selection in order to generate comparable follow-up time periods as the cases.

### Study variables

The social security system registers the date of death as long as the individual is in contact with the social security, which was used to determine case or control status. Three indicators of employment history were examined: employment, unemployment and inactivity. Employment was defined as working with either a legal job contract or being registered as self-employed in the social security system. Unemployment referred to a period in which a subject was receiving unemployment benefits from the social security system. Inactivity consisted of a period during which an individual was not in contact with the social security system, which indicates the individual is not employed formally nor is receiving any unemployment benefits. For each of these three broad indicators, we constructed two indicators. One measured the time spent (in months) in a particular situation, and the other indicator measured the number of times a person entered the situation over their working life. The time in each situation measures the magnitude of the “exposure”. The number of times entering a situation reflects the stability of the working life. Time employed was determined by the start and end dates of a job contract or self-employment period; time unemployed by the start and end dates of receipt of unemployment benefits, and time in inactivity referred to the time an individual was not in contact with the social security system but had administrative activity before and after the inactive period. Time in inactivity was only counted if it was greater than 30 days, in order to exclude time lags between transitions from one administrative situation to another (e.g. from one job to another, from employment to unemployment with benefits, from employment to retirement pension). Each indicator was defined as both a continuous and discrete variable. Continuous variables were transformed to natural log due to their non-normal distribution, and discrete variables were classified into tertiles.

Covariates included permanent disability and occupational category. Permanent disability refers to the pension that the social security grants to workers whose illness or injury impedes their continued participation in the labor force if they have not reached retirement age. We assigned this state to persons who left the workforce due to a permanent disability pension before age 65. Occupational category was constructed from the professional category reported by the employer, which refers to the physical demands and qualifications required for a job. Occupational category is known to be related to health [19]. For a sensitivity analysis we constructed the variable labor relationship, to identify temporary, permanent and self-employed workers. Since individuals may have several occupations over a lifetime, the occupational category and the assigned labor relationship were defined by the longest period of time spent in each of these states.

### Statistical analysis

We used logistic regression to measure the associations between the three employment history indicators and case or control status, while adjusting for permanent disability and occupational category, including those with uninformed occupational category. Regressions were performed separately by state, we did not include the three indicators in the same model. Analyses were conducted separately for women and men given the gender differences in labor force participation in Spain [20]. Logistic regressions were conducted using the variables in their continuous and discrete forms to look at trends and to better understand the magnitude of any associations observed. To test the consistency of the results, four separate sensitivity analyses were performed. First, we limited the analysis to cases and controls under 65 years of age to look at premature deaths. Next, we repeated the analysis among individuals with more than 19 years with a registered employment history to account for similar times of follow-up. Then, two stratified analysis were performed: one by occupational category, adjusted only by permanent disability, and another one by type of labor relationship (permanent, temporary and self-employed) given that these two factors are known to have an effect on health [19].

## Results

Between 2004 and 2013, there were 53,989 deaths in the WORKss cohort (men: 42,550; women: 11,439). After controls were matched, the sample doubled to 107,978 individuals, with a median age of 74 (P25:62, P75:80) and 72 (P25:62, P75:78) years for women and men, respectively. More than 70% of the sample consisted of persons aged 65 and over and with a permanent labor relationship. Almost 80% of individuals (n = 84,897) had 9 years of follow-up information available during the working age (Table 1). There were differences with respect to the proportion of cases and controls who had a permanent disability in both women (cases: 33%, controls: 17%) and men (cases: 31%, controls: 16%).

**Table 1:** Characteristics of employment history indicators: Employment, unemployment and Inactivity, by sex of cases and controls in the Spanish WORKing Life Social Security (WORKss) cohort study 2004–2013.

		Women				Men			
		Controls (n = 11,439)		Cases (n = 11,439)		Controls (n = 42,550)		Cases (n = 42,550)	
		No.	%	No.	%	No.	%	No.	%
**Employment**									
** Months**									
Continuous:	Median [P25,P75]	140 [75,189]		131 [65,181]		170 [114,255]		152 [95,226]	
Discrete:	0–121 (0–9 years)	4,557	39.8	5,086	44.5	11,728	27.6	147,24	34.6
	122–197 (10–16 years)	4,597	40.2	4,376	38.3	14,816	34.8	14,982	35.2
	>197 (>16 years)	2,285	20	1,978	17.3	16,006	37.6	12,844	30.2
** Number of contracts**									
Continuous:	Median [P25,P75]	2 [1,4]		2 [1,4]		2 [1,5]		2 [1,6]	
Discrete:	1	5,096	44.6	5,158	45.1	14,171	33.3	14,070	33.1
	2–4	3,835	33.5	3,871	33.8	15,712	36.9	15,335	36
	>4	2,508	21.9	2,411	21.1	12,667	29.8	13,145	30.9
**Unemployment**									
** Months**									
Continuous:	Median [P25,P75]	0 [0,6]		0 [0,5]		0 [0,23]		0 [0,24]	
Discrete:	0	8,135	71.1	8,243	72.1	23,497	55.2	22,434	52.7
	1–12	922	8.1	797	7	4,254	10	3,984	9.4
	>12	2,382	20.8	2,400	21	14,799	34.8	16,132	37.9
** Number of spells**									
Continuous:	Median [P25,P75]	0 [0,1]		0 [0,1]		0 [0,2]		0 [0,2]	
Discrete:	0	8,079	70.6	8,181	71.5	22,933	53.9	21,981	51.7
	1	1,125	9.8	955	8.4	6,840	16.1	6,376	15
	>1	2,235	19.5	2,304	20.1	12,777	30	14,193	33.4
**Inactivity**									
** Months**									
Continuous:	Median [P25,P75]	0 [0,15]		0 [0,17]		0 [0,9]		0 [1,16]	
Discrete:	0	6,579	57.5	6,463	56.5	23,012	54.1	20,980	49.3
	1–5	1,220	10.7	1,244	10.9	6,962	16.4	6,511	15.3
	>6	3,640	31.8	3,733	32.6	12,576	29.6	15,059	35.4
** Number of spells**									
Continuous:	Median [P25,P75]	0 [0,1]		0 [0,1]		0 [0,1]		0 [0,2]	
Discrete:	0	6,731	58.8	6,582	57.5	24,402	57.4	22,048	51.8
	1	2,067	18.1	2,176	19	7,801	18.3	7,863	18.5
	>1	2,641	23.1	2,682	23.4	10,347	24.3	12,639	29.7
**Labor relationship**									
Permanent		8,678	75.9	8,879	77.6	33,985	79.9	34,779	81.7
Temporary		334	2.9	337	3	1,006	2.4	878	2.1
Self-employed		2,427	21.2	2,223	19.4	7,559	17.8	6,893	16.2
**Occupational category**									
Skilled non-manual		754	7	649	6	5,221	12	4,021	10
Skilled manual		1,282	11.2	1,305	11.4	14,181	33.3	14,574	34.3
Unskilled non-manual		1,920	16.8	1,895	16.6	6,692	15.7	6,346	14.9
Unskilled manual		3,210	28.1	3,411	29.8	9,293	21.8	11,276	26.5
Self-employed		1,640	14.3	1,526	13.3	3,679	8.7	3,276	7.7
Not informed		2,633	23	2,654	23.2	3,484	8.2	3,057	7.2
**Permanent disability**									
No		9,460	82.7	7,656	66.9	35,615	83.7	29,227	68.7
Yes		1,979	17.3	3,784	33.1	6,935	16.3	13,323	31.3
**Years of follow-up**									
0–9		3,177	27.8	3,613	31.6	7,409	17.4	8,882	20.9
10–19		5,721	50	5,542	48.5	19,380	45.6	19,836	46.6
>19		2,541	22.2	2,284	20	15,761	37	13,832	32.5

Table 2 presents the odds ratios adjusted by permanent disability and occupational category for all three employment history indicators. More time in employment (>16 years) showed a significant inverse association with mortality in both women (odds ratio = 0.88; 95% confidence interval: 0.81, 0.94) and men (odds ratio = 0.76; 95% confidence interval: 0.74, 0.79). Among men, more spells of unemployment (>1 spell OR: 1.14; 95% confidence interval: 1.10, 1.18) and of inactivity (>1 spell, OR: 1.24; 95% confidence interval: 1.19, 1.28), as well as more time spent in either unemployment (>12 months, OR: 1.16; 95% confidence interval: 1.12, 1.20) or inactivity (>6 months, OR: 1.17; 95% confidence interval: 1.14, 1.21) were significant predictors of mortality; a similar relationship was not observed in women. Sensitivity analyses conducted on a subset of cases under age 65 and their controls showed similar results for men, as well as a significant relationship between total number of contracts over the working life and mortality (Table 3). Except for a protective association of more time spent in employment, no other significant associations were found in women.

**Table 2:** Association (Odds ratio = OR) of mortality and employment history indicators: Employment, unemployment and inactivity in the Spanish WORKing Life Social Security (WORKss) cohort study 2004–2013.

		Women				Men		
	OR^a^	95% CI^b^	OR^c^	95% CI^b^	OR^a^	95% CI^b^	OR^c^	95% CI^b^
**Employment**								
** Months**								
1. Continuous	0.96	0.94, 0.98*	0.96	0.95, 0.98*	0.85	0.83, 0.86*	0.92	0.91, 0.94*
2. Discrete categories								
0–121 (0–9 years)	1.00		1.00		1.00		1.00	
122–197 (10–16 years)	0.85	0.81, 0.90*	0.94	0.89, 1.00	0.81	0.78, 0.83*	0.94	0.91, 0.97*
>197 (>16 years)	0.78	0.72, 0.83*	0.88	0.81, 0.94*	0.64	0.62, 0.66*	0.76	0.74, 0.79*
** Number of contracts**								
3. Continuous	0.98	0.96, 1.01	0.97	0.94, 1.00*	1.04	1.02, 1.05*	1.02	1.01, 1.04*
4. Discrete categories								
1	1.00		1.00		1.00		1.00	
2–4	0.99	0.94, 1.06	0.96	0.90, 1.02	0.98	0.95, 1.02	0.95	0.92, 0.99*
>4	0.95	0.89, 1.02	0.91	0.84, 0.99*	1.05	1.01, 1.08*	1.00	0.96, 1.04
**Unemployment**								
** Months**								
5. Continuous	1.00	0.99, 1.00	1.00	0.99, 1.00	1.01	1.01, 1.01*	1.01	1.01, 1.01*
6. Discrete categories								
0	1.00		1.00		1.00		1.00	
1–12	0.85	0.77, 0.94*	0.84	0.76, 0.94*	0.98	0.94, 1.03	0.95	0.91, 1.00
>12	0.99	0.93, 1.06	1.00	0.93, 1.08	1.14	1.11, 1.18*	1.16	1.12, 1.20*
** Number of spells**								
7. Continuous	1.00	0.99, 1.00	1.00	0.99, 1.00	1.01	1.01, 1.02*	1.01	1.01, 1.02*
8. Discrete categories								
0	1.00		1.00		1.00		1.00	
1	0.84	0.76, 0.92*	0.84	0.76, 0.93*	0.97	0.94, 1.01*	1.01	0.97, 1.06
>1	1.02	0.95, 1.09	1.02	0.95, 1.10	1.16	1.12, 1.19*	1.14	1.10, 1.18*
**Inactivity**								
** Months**								
9. Continuous	1.00	1.00, 1.01	1.00	1.00, 1.00	1.02	1.02, 1.03*	1.01	1.01, 1.02*
10. Discrete categories								
0	1.00		1.00		1.00		1.00	
1–5	1.04	0.95, 1.13	0.98	0.89, 1.07	1.03	0.99, 1.07	0.99	0.95, 1.03
>6	1.04	0.99, 1.11	0.98	0.92, 1.04	1.31	1.27, 1.35*	1.17	1.14, 1.21*
** Number of spells**								
11. Continuous	1.01	1.00, 1.01*	1.00	0.99, 1.01	1.03	1.03, 1.03*	1.02	1.02, 1.02*
12. Discrete categories								
0	1.00		1.00		1.00		1.00	
1	1.08	1.00, 1.15*	0.98	0.92, 1.06	1.12	1.08, 1.16*	1.02	0.98, 1.06
>1	1.04	0.97, 1.11	0.99	0.93, 1.07	1.35	1.31, 1.40*	1.24	1.19, 1.28*

^a^ Unadjusted odd ratios

^b^Confidence Interval

^c^ Adjusted for permanent disability and occupational category

* p-value <0.05

**Table 3:** Association (Odds ratio = OR) of mortality before sge 65 and labor trajectory indicators: Employment, unemployment and inactivity by sex for cases and controls in the Spanish WORKing Life Social Security (WORKss) Cohort Study 2004–2013.

	Women				Men			
	OR^a^	95% CI^b^	OR^c^	95% CI^b^	OR^a^	95% CI^b^	OR^c^	95% CI^b^
**Employment**								
** Months**								
Continuous	0.94	0.90, 0.98*	0.88	0.84, 0.92*	0.75	0.73, 0.78*	0.82	0.80, 0.85*
Discrete categories								
0–121 (0–9 years)	1.00		1.00		1.00		1.00	
122–197 (10–16 years)	0.86	0.76, 0.98*	0.71	0.62, 0.81*	0.88	0.81, 0.96*	0.83	0.76, 0.91*
>197 (>16 years)	0.75	0.67, 0.84*	0.71	0.63, 0.80*	0.54	0.51, 0.58*	0.64	0.60, 0.69*
** Number of contracts**								
Continuous	0.98	0.93, 1.02	0.98	0.92, 1.03	1.11	1.09, 1.14*	1.12	1.09, 1.15*
Discrete categories								
1	1.00		1.00		1.00		1.00	
2–4	1.02	0.88, 1.18	0.98	0.83, 1.17	0.96	0.88, 1.05	0.99	0.90, 1.10
>4	0.96	0.84, 1.11	0.95	0.80, 1.13	1.10	1.02, 1.20*	1.13	1.02, 1.25*
**Unemployment**								
** Months**								
Continuous	1.00	1.00, 1.01	1.00	1.00, 1.01	1.03	1.02, 1.03*	1.02	1.01, 1.02*
Discrete categories								
0	1.00		1.00		1.00		1.00	
1–12	0.91	0.79, 1.04	0.96	0.82, 1.12	0.99	0.92, 1.06	1.00	0.92, 1.08
>12	1.10	0.99, 1.22	1.06	0.94, 1.21	1.41	1.34, 1.49*	1.26	1.18, 1.33*
** Number of spells**								
Continuous	1.00	0.99, 1.02	1.00	1.00, 1.02	1.03	1.02, 1.04*	1.02	1.01, 1.03*
Discrete categories								
0	1.00		1.00		1.00		1.00	
1	0.99	0.85, 1.15	0.99	0.84, 1.18	0.97	0.90, 1.05	0.98	0.90, 1.06
>1	1.07	0.96, 1.19	1.07	0.95, 1.21	1.36	1.28, 1.43*	1.22	1.15, 1.29*
**Inactivity**								
** Months**								
Continuous	1.01	1.00, 1.02*	1.00	1.00, 1.02	1.04	1.04, 1.05*	1.03	1.03, 1.04*
Discrete categories								
0	1.00		1.00		1.00		1.00	
1–5	1.00	0.84, 1.18	0.95	0.79, 1.15	1.05	0.97, 1.13	0.99	0.92, 1.08
>6	1.13	1.01, 1.26*	1.07	0.94, 1.21	1.60	1.51, 1.69*	1.41	1.32, 1.50*
** Number of spells**								
Continuous	1.01	1.00, 1.03*	1.01	1.00, 1.02	1.06	1.05, 1.06*	1.04	1.03, 1.05*
Discrete categories								
0	1.00		1.00		1.00		1.00	
1	1.12	0.97, 1.29	1.04	0.89, 1.21	1.20	1.12, 1.29*	1.12	1.04, 1.21*
>1	1.13	1.02, 1.26*	1.09	0.96, 1.23	1.65	1.56, 1.74*	1.45	1.37, 1.55*

^a^ Unadjusted odd ratios

^b^Confidence Interval

^c^ Adjusted for permanent disability and occupational category

* p-value <0.05

Sensitivity analyses by labor relationship (Table 4 for temporary workers and S1 and S2 Tables for permanent and self-employed workers), by occupational category (S3–S6 Tables) and for persons with more than 19 years of follow-up (S7 Table) showed similar trends in results to those in the overall population. However, the mortality risk for men who mostly worked under temporary contracts (n = 1,884) was significantly higher in the presence of an increasing number of these types of contracts (>4 contracts, odds ratio = 1.39, 95% confidence interval: 1.02, 1.90). In this subgroup, there were also much stronger associations of months in unemployment (>12 months in unemployment, odds ratio = 1.42, 95% confidence interval: 1.13, 1.78) and months spent in inactivity (>6 months in inactivity, odds ratio = 1.85, 95% confidence interval 1.47, 2.33) than those of the overall sample. Results by occupational category show that inactivity is more detrimental to manual categories irrespective of skill level, but trends for the other factors were similar to the whole sample results in all the categories.

**Table 4:** Association (Odds ratio = OR) of mortality and labor trajectory indicators: Employment, unemployment and inactivity in temporary workers in the Spanish WORKing Life Social Security (WORKss) Cohort Study 2004–2013.

	Women				Men			
	OR^a^	95% CI^b^	OR^c^	95% CI^b^	OR^a^	95% CI^b^	OR^c^	95% CI^b^
**Employment**								
** Months**								
Continuous	0.94	0.87, 1.03	0.92	0.85, 1.01	0.94	0.89, 0.99*	0.96	0.91, 1.01
Discrete categories								
0–121 (0–9 years)	1.00		1.00		1.00		1.00	
122–197 (10–16 years)	0.87	0.58, 1.32	0.77	0.49, 1.20	1.09	0.82, 1.47	1.02	0.75, 1.38
>197 (>16 years)	0.61	0.39, 0.96*	0.64	0.40, 1.03	0.53	0.43, 0.65*	0.60	0.47, 0.76*
** Number of contracts**								
Continuous	1.00	0.87, 1.15	0.98	0.85, 1.13	1.26	1.16, 1.37*	1.20	1.10, 1.30*
Discrete categories								
1	1.00		1.00		1.00		1.00	
2–4	1.36	0.77, 2.40	1.26	0.70, 2.26	1.19	0.85, 1.66	1.19	0.85, 1.67
>4	1.14	0.68, 1.90	1.06	0.62, 1.81	1.60	1.19, 2.17*	1.39	1.02, 1.90*
**Unemployment**								
** Months**								
Continuous	1.00	0.97, 1.03	0.99	0.96, 1.02	1.04	1.02, 1.06*	1.02	1.00, 1.04*
Discrete categories								
0	1.00		1.00		1.00		1.00	
1–12	0.88	0.60, 1.29	0.86	0.57, 1.28	1.16	0.92, 1.46	1.02	0.80, 1.29
>12	1.06	0.75, 1.51	0.96	0.66, 1.38	1.75	1.41, 2.16*	1.42	1.13, 1.78*
** Number of spells**								
Continuous	1.00	0.97, 1.04	0.99	0.96, 1.03	1.05	1.02, 1.07*	1.03	1.00, 1.05*
Discrete categories								
0	1.00		1.00		1.00		1.00	
1	1.08	0.68, 1.71	1.02	0.63, 1.65	0.93	0.70, 1.24	0.85	0.63, 1.14
>1	0.98	0.71, 1.36	0.93	0.66, 1.30	1.62	1.33, 1.97*	1.34	1.09, 1.65*
**Inactivity**								
** Months**								
Continuous	1.01	0.97, 1.04	1.00	0.96, 1.03	1.08	1.06, 1.10*	1.06	1.04, 1.08*
Discrete categories								
0	1.00		1.00		1.00		1.00	
1–5	0.45	0.25, 0.82*	0.39	0.21, 0.72*	1.14	0.84, 1.57	1.07	0.78, 1.47
>6	1.05	0.71, 1.54	0.95	0.64, 1.42	2.21	1.78, 2.76*	1.85	1.47, 2.33*
** Number of spells**								
Continuous	1.01	0.97, 1.04	1.00	0.96, 1.03	1.08	1.06, 1.10*	1.06	1.04, 1.08*
Discrete categories								
0	1.00		1.00		1.00		1.00	
1	0.88	0.52, 1.50	0.87	0.50, 1.51	1.51	1.11, 2.04*	1.41	1.03, 1.92*
>1	1.02	0.70, 1.48	0.93	0.63, 1.38	2.25	1.82, 2.79*	1.89	1.51, 2.37*

^a^ Unadjusted odd ratios

^b^Confidence Interval

^c^ Adjusted for permanent disability and occupational category

* p-value <0.05

## Discussion

To our knowledge, this is the first study examining employment history indicators and mortality in a large working population sample. For both men and women, employment and working longer protected against premature (before age 65) and overall mortality. However, there was a differential association of work in women as compared to men. Findings about unstable work for men are in line with expectations: unemployment and, particularly, inactivity were associated with an increased risk of death [8, 9, 21, 22]. These associations were stronger for men in a temporary labor relationship. In contrast, neither unemployment nor any indicator of inactivity showed a similar pattern in women. In fact, having a greater number of contracts over the working life appeared to be protective in women. Previous studies on time spent working, without considering working conditions, found that women and men who work longer or are continuously employed have better health [8, 22–25]. Our findings are in line with these results, even for temporary workers, despite some previous studies showing worse morbidity and mortality outcomes in this latter group [16, 26–28]. On the other hand, stronger relationships (greater mortality risk), for temporary workers regarding unemployment and inactivity as compared to the overall sample may be explained by factors related to temporary employment such as job insecurity, precariousness, lower salaries and higher stress levels [26, 29].

We also assessed if a flexible working life measured by the number of contracts had an association with mortality. In the overall study population, the number of contracts in a labor trajectory was not related to a greater odds of mortality. However, for persons under 65 years of age and temporary workers, the greater the number of contracts over a working life, the greater the risk of death. This may be explained in two not mutually exclusive ways. First, due to the healthy worker effect on workers with few contracts, there is a lack of relationship between number of contracts and overall mortality. Those selected into work obtain better, longer-lasting contracts [30]. In contrast, those who are ill or not as healthy may have more contracts due to frequent job changes because of their health. Second, the rise in temporary employment in Spain since the late 1980s may explain why younger cohorts are more exposed to a greater number of contracts over a lifetime: 7% of the younger cohort had a temporary labor relationship compared to less than 1% for those age 64 and older. In this sense, results regarding number of contracts should be interpreted carefully since one limitation was that we do not have access to baseline health. However, analyses were adjusted for permanent disability, which is always granted after one or more temporary sickness absence episodes [31]. To a certain extent, then, we take into account previous health problems when we adjust for this variable, although we need to continue studying this effect in the future.

In comparison to other studies examining the working life course, we included inactivity, which provides a broader perspective of working lives. The relationships between mortality and time spent in inactivity (>6months, odds ratio = 1.17) and unemployment (>12 months, odds ratio = 1.16) were similar in men. One of the limitations in our study is that we do not know the actual activity of individuals during their “inactivity” period. This is reflected by an administrative gap, which we call inactivity. During this period, persons could be working in a different country, but they could also be working in the informal economy, be unemployed without benefits or retired without pension, factors detrimental to health [32]. In fact, results show that the risk of death increased at an earlier point in time with inactivity (≥7 months) than it did with unemployment (≥12 months), suggesting inactivity is a more vulnerable state. There may be a differential regarding activity during inactive administrative gaps by occupational category. S3–S6 Tables show that inactivity is more detrimental to men in manual categories irrespective of skill which may reflect increased vulnerability for these groups in situations of inactivity. It would not be surprising to see similar or greater risks in a context where unemployment benefits are not as generous [33], not granted during long enough periods or involve more restrictive eligibility criteria.

An important feature of this study is the assessment of employment history indicators, separated by sex. For women, results were inconclusive; more time in unemployment and inactivity was not related to higher mortality. Male labor force participation rates in Spain were stable at around 80% from 1981 to 2014 while female rates in the 1980s were as low as 30% but gradually increased to 69% during the first years of the 21st century [20]. Given the mean age of our sample, most labor activity occurred during the 1980s and 1990s, i.e., at a time of low labor participation among women. Low participation rates reflect women´s role in society then. Evidence from Spain and elsewhere show that, most often, men are the main breadwinners of the household and women the main homemakers [34, 35]. For this reason, being gainfully involved in the labor force may have more of an effect on men´s health than on women´s health, where the effect of their non-remunerated role at home on health would not have been detected. A study assessing unemployment and mental health in a Catalonian (Spain) population sample found different associations according to gender and family roles. Married men and single women who experienced unemployment had worse mental health than their counterparts (married women and single men), and results differed by social class [34]. Even though this study did not focus on mortality, it showed that the relation between work and health depends significantly on family roles related to economic provision for the household. Individuals who are not the main economic providers of the household are less affected by employment instability than individuals whose main role is that of the breadwinner. In our study, one limitation is the lack of information regarding family characteristics, and our results may reflect that women in contact with the social security at the time are most likely not the main breadwinners.

Several studies looking at characteristics of working lives and morbidity or mortality have found similar results regarding differences between males and females [6, 16]. However, there is literature that shows that unemployment is also detrimental to women´s health [8, 36]. Even so, the effect is usually stronger in men [37], confirming their main breadwinner role. An exception was found in a study of labor trajectories and mortality conducted in the Wisconsin Longitudinal study [24]. Women´s labor trajectories, characterized by either entering or exiting the labor market at midlife age, were associated with mortality after adjusting for health status, economic circumstances, marital status and employment characteristics. In men, unstable trajectories were not associated with mortality. It should be noted, though, that female labor participation in the US was much higher (60%) in the 1980s and 1990s than in Spain, and the methods included a latent class analysis approach using days worked per year rather than separate labor trajectory indicators. Also, it has been suggested that worse health in the US is related to barriers in accessing healthcare [24], with access to health care and pension benefits being dependent on employment [23, 24]. In contrast, in Spain access to health care in the 1980s and 1990s was universal and only pensions were job-related [10].

Using the WORKss cohort to analyze employment history gave us the advantage of covering a long follow-up period, 32 years, and access to information on a variety of workers in different economic activities. WORKss provides administrative data from Spain that include records of each contract and each period receiving unemployment, retirement or permanent disability pension benefits, limiting recall bias or inaccuracies that may occur with self-reported data [38, 39]. Few studies have assessed the association of employment history on health and usually covered follow-up periods shorter than 10 years [3] or had data from few self-reported waves rather than accurate date by date information on labor relationships [24]. Furthermore, we were able to tease out the role of inactivity from unemployment in the social security system. Inactivity may represent a situation of low economic protection, a situation not previously studied. Moreover, the association of unemployment on mortality could be underestimated because unemployed workers in our sample are receiving a subsidy. Recent research suggests that unemployment benefits reduce the negative health effects of unemployment [33].

Our findings are based on a representative sample of members registered in the social security system at some point between 2004 and 2013. In this sense, we are studying a privileged sample that “survived” until 2004. Thus, results may be underestimated since they reflect the detrimental effects of unemployment and inactivity in a sample that has been at the core of the labor market, rather than at the periphery for both controls and cases. This is a limitation because we do not have information on those who died while not in contact with the social security. However, when members start receiving their retirement pension at age 65, the social security does not lose information regarding death; so most likely those who die without contact with social security either were not able to work long enough to be eligible for a retirement pension or died during inactivity periods before reaching retirement age.

Case-control samples matched on age are advantageous because many labor market situations are related to age, and age is related to mortality. For example, unemployment spells do not affect individuals the same way at younger ages than at older ages [40, 41]. Context is also important; cases and controls are in the labor market at the same periods of time, hence they experience economic downturns and periods of economic growth at the same time and age [17]. Even though, age is highly correlated to employment history indicators focused on time in each situation and mortality, results of odds ratios controlling for age did not show different results as those presented in this study. Nevertheless, by matching on age, we may be missing the effect of unmeasured variables related to age.

Results in this study highlight the importance of employment during working age and its association with mortality. Time employed was protective against mortality in a population whose working lives occurred mostly during the last decades of the twentieth century and remained in contact with the social security system from 2004 to 2013. Cumulative unemployment and inactivity were detrimental to health for men. Nevertheless, findings show that gaps in employment were not relevant to women who formed part of the labor force at the time. However, with societal, cultural, economic and political changes, women´s roles in society have changed. Women are pursuing professional careers, becoming main breadwinners, and in general forming different family types. These aspects make women, nowadays, as dependent on work as men [35]. Thus, the population at risk of morbidity and mortality due to unstable employment is increasing [42]. There should be further assessment of the double burden women carry at home and work, plus family policies that ease the roles in the household and at work. For this reason, it is important to continue studying the effect of recent labor trajectories on health in both women and men utilizing methods that summarize work from a life course perspective.

We consider our results are very relevant given the context of recent demographic, policy and labor market changes. There is growing concern related to the rapid expansion of the older population in contrast with the younger one. This change requires a young labor force that provides enough contributions to sustain social benefits. If the labor market is shifting towards flexible employment, our results show that special attention should be paid to unemployment and inactivity periods. Results also reflect the need for further research to understand the dynamics of the labor market and social protections. The structure of this symbiosis may be key to overcoming the negative effects of changes in the labor market.

## Supporting information

S1 Table Association (Odds ratio = OR) of mortality and employment history indicators: Employment, unemployment and inactivity in permanent workers in the Spanish WORKing Life Social Security (WORKss) cohort study 2004–2013. ^a^ Unadjusted odd ratios. ^b^Confidence Interval. ^c^ Adjusted for permanent disability and occupational category. * p-value <0.05
(DOCX)

S2 Table Association (Odds ratio = OR) of mortality and employment history indicators: Employment, unemployment and inactivity in self-employed workers in the Spanish WORKing Life Social Security (WORKss) cohort study 2004–2013. ^a^ Unadjusted odd ratios. ^b^Confidence Interval. ^c^ Adjusted for permanent disability and occupational category. * p-value <0.05
(DOCX)

S3 Table Association (Odds ratio = OR) of mortality and labor trajectory indicators: Employment, Unemployment and Inactivity in Skilled Non-manual Workers in the Spanish WORKing Life Social Security (WORKss) Cohort Study 2004–2013. ^a^ Unadjusted odd ratios. ^b^Confidence Interval. ^c^ Adjusted for permanent disability and occupational category. * p-value <0.05
(DOCX)

S4 Table Association (Odds ratio = OR) of mortality and labor trajectory indicators: Employment, unemployment and inactivity in skilled manual workers in the Spanish WORKing Life Social Security (WORKss) cohort study 2004–2013. ^a^ Unadjusted odd ratios. ^b^Confidence Interval. ^c^ Adjusted for permanent disability and occupational category. * p-value <0.05
(DOCX)

S5 Table Association (Odds ratio = OR) of mortality and labor trajectory indicators: Employment, unemployment and inactivity in unskilled non-manual workers in the Spanish WORKing Life Social Security (WORKss) cohort study 2004–2013. ^a^ Unadjusted odd ratios. ^b^Confidence Interval. ^c^ Adjusted for permanent disability and occupational category. * p-value <0.05
(DOCX)

S6 Table Association (Odds ratio = OR) of mortality and labor trajectory indicators: Employment, unemployment and inactivity in unskilled manual workers in the Spanish WORKing Life Social Security (WORKss) cohort study 2004–2013. ^a^ Unadjusted odd ratios. ^b^Confidence Interval. ^c^ Adjusted for permanent disability and occupational category. * p-value <0.05.
(DOCX)

S7 Table Association (Odds ratio = OR) of mortality and employment history indicators: Employment, unemployment and inactivity in individuals with over 19 years of follow-up time in the Spanish WORKing Life Social Security (WORKss) cohort study 2004–2013. ^a^ Unadjusted odd ratios. ^b^Confidence Interval. ^c^ Adjusted for permanent disability and occupational category. * p-value <0.05
(DOCX)
