# Supplementary material for: Employment history indicators and mortality in a nested case-control study from the Spanish WORKing life social security (WORKss) cohort
Source: PLoS One. 2017 Jun 1;12(6):e0178486. doi: 10.1371/journal.pone.0178486 (PMC5453531; doi:10.1371/journal.pone.0178486)
Supplement: S2 Table — a Unadjusted odd ratios. bConfidence Interval. c Adjusted for permanent disability and occupational category. * p-value <0.05 (DOCX) [file pone.0178486.s002.docx]

|  | | |  | **Women** | | | |  | **Men** | | | |
| --- | --- | --- | --- | --- | --- | --- | --- | --- | --- | --- | --- | --- |
|  | | |  | **OR^a^** | **95% CI^b^** | **OR^c^** | **95% CI^b^** |  | **OR^a^** | **95% CI^b^** | **OR^c^** | **95% CI^b^** |
| **Employment** | | | |  |  |  |  |  |  |  |  |  |
|  | **Months** | | |  |  |  |  |  |  |  |  |  |
|  | | Continuous | | 0.88 | 0.82, 0.94* | 0.92 | 0.85, 0.99 * |  | 0.75 | 0.71, 0.80 * | 0.85 | 0.80, 0.90* |
|  | | Discrete categories | |  |  |  |  |  |  |  |  |  |
|  | | 0-121 (0-9 years) | | 1.00 |  | 1.00 |  |  | 1.00 |  | 1.00 |  |
|  | | 122-197 (10-16 years) | | 0.81 | 0.71, 0.93* | 0.92 | 0.80, 1.07 |  | 0.82 | 0.75, 0.91 * | 1.02 | 0.92, 1.13 |
|  | | >197 (>16 years) | | 0.73 | 0.62, 0.85* | 0.84 | 0.72, 0.99 * |  | 0.64 | 0.58, 0.70 * | 0.79 | 0.71, 0.87* |
|  | **Number of contracts** | | |  |  |  |  |  |  |  |  |  |
|  | | Continuous | | 0.91 | 0.84, 0.99* | 0.86 | 0.76, 0.97 * |  | 1.02 | 0.99, 1.07 | 1.00 | 0.94, 1.07 |
|  | | Discrete categories | |  |  |  |  |  |  |  |  |  |
|  | | 1 | | 1.00 |  | 1.00 |  |  | 1.00 |  | 1.00 |  |
|  | | 2-4 | | 0.87 | 0.77, 0.99* | 0.84 | 0.72, 0.98 * |  | 1.00 | 0.93, 1.07 | 0.93 | 0.83, 1.04 |
|  | | >4 | | 0.86 | 0.69, 1.07 | 0.74 | 0.56, 0.99 * |  | 1.02 | 0.93, 1.12 | 0.90 | 0.78, 1.05 |
| **Unemployment** | | | |  |  |  |  |  |  |  |  |  |
|  | **Months** | | |  |  |  |  |  |  |  |  |  |
|  | | Continuous | | 0.99 | 0.97, 1.01 | 0.99 | 0.96, 1.01 |  | 1.01 | 1.00, 1.02 * | 1.01 | 1.00, 1.02 |
|  | | Discrete categories | |  |  |  |  |  |  |  |  |  |
|  | | 0 | | 1.00 |  | 1.00 |  |  | 1.00 |  | 1.00 |  |
|  | | 1-12 | | 1.01 | 0.75, 1.37 | 0.94 | 0.67, 1.34 |  | 0.97 | 0.86, 1.08 | 0.94 | 0.82, 1.07 |
|  | | >12 | | 0.87 | 0.67, 1.13 | 0.85 | 0.61, 1.16 |  | 1.20 | 1.09, 1.32 * | 1.15 | 1.02, 1.30* |
|  | **Number of spells** | | |  |  |  |  |  |  |  |  |  |
|  | | Continuous | | 0.99 | 0.97, 1.02 | 0.99 | 0.96, 1.02 |  | 1.01 | 1.00, 1.02 * | 1.01 | 0.99, 1.02 |
|  | | Discrete categories | |  |  |  |  |  |  |  |  |  |
|  | | 0 | | 1.00 |  | 1.00 |  |  | 1.00 |  | 1.00 |  |
|  | | 1 | | 1.06 | 0.78, 1.44 | 0.99 | 0.70, 1.41 |  | 0.95 | 0.85, 1.06 | 0.94 | 0.82, 1.07 |
|  | | >1 | | 0.90 | 0.70, 1.16 | 0.92 | 0.98, 1.26 |  | 1.19 | 1.08, 1.31 * | 1.12 | 1.00, 1.26 |
| **Inactivity** | | | |  |  |  |  |  |  |  |  |  |
|  | **Months** | | |  |  |  |  |  |  |  |  |  |
|  | | Continuous | | 1.00 | 0.99, 1.02 | 1.00 | 0.99, 1.02 |  | 1.02 | 1.01, 1.02 * | 1.01 | 1.00, 1.02* |
|  | | Discrete categories | |  |  |  |  |  |  |  |  |  |
|  | | 0 | | 1.00 |  | 1.00 |  |  | 1.00 |  | 1.00 |  |
|  | | 1-5 | | 0.98 | 0.77, 1.23 | 0.93 | 0.73, 1.19 |  | 1.06 | 0.96, 1.17 | 1.03 | 0.92, 1.15 |
|  | | >6 | | 1.06 | 0.92, 1.21 | 1.03 | 0.87, 1.21 |  | 1.22 | 1.13, 1.32 * | 1.16 | 1.05, 1.28* |
|  | **Number of spells** | | |  |  |  |  |  |  |  |  |  |
|  | | Continuous | | 1.00 | 0.99, 1.02 | 1.00 | 0.99, 1.02 |  | 1.02 | 1.01, 1.02 * | 1.01 | 1.01, 1.02* |
|  | | Discrete categories | |  |  |  |  |  |  |  |  |  |
|  | | 0 | | 1.00 |  | 1.00 |  |  | 1.00 |  | 1.00 |  |
|  | | 1 | | 1.04 | 0.89, 1.21 | 0.99 | 0.85, 1.17 |  | 1.14 | 1.05, 1.24 * | 1.10 | 1.00, 1.22 |
|  | | >1 | | 1.05 | 0.88, 1.25 | 1.04 | 0.82, 1.30 |  | 1.23 | 1.13, 1.34 * | 1.19 | 1.07, 1.33* |
