# Supplementary material for: Employment history indicators and mortality in a nested case-control study from the Spanish WORKing life social security (WORKss) cohort
Source: PLoS One. 2017 Jun 1;12(6):e0178486. doi: 10.1371/journal.pone.0178486 (PMC5453531; doi:10.1371/journal.pone.0178486)
Supplement: S4 Table — a Unadjusted odd ratios. bConfidence Interval. c Adjusted for permanent disability and occupational category. * p-value <0.05 (DOCX) [file pone.0178486.s004.docx]

|  | | |  | **Women** | | | |  | **Men** | | | |
| --- | --- | --- | --- | --- | --- | --- | --- | --- | --- | --- | --- | --- |
|  | | |  | **OR^a^** | **95% CI^b^** | **OR^c^** | **95% CI^b^** |  | **OR^a^** | **95% CI^b^** | **OR^c^** | **95% CI^b^** |
| **Employment** | | | |  |  |  |  |  |  |  |  |  |
|  | **Months** | | |  |  |  |  |  |  |  |  |  |
|  | | Continuous | | 0.96 | 0.91, 1.01 | 0.94 | 0.89, 0.99* |  | 0.84 | 0.81, 0.86 | 0.87 | 0.84, 0.90* |
|  | | Discrete categories | |  |  |  |  |  |  |  |  |  |
|  | | 0-121 (0-9 years) | | 1.00 |  | 1.00 |  |  | 1.00 |  | 1.00 |  |
|  | | 122-197 (10-16 years) | | 0.92 | 0.77, 1.10 | 0.88 | 0.74, 1.06 |  | 0.85 | 0.80, 0.90* | 0.89 | 0.84, 0.95* |
|  | | >197 (>16 years) | | 0.82 | 0.67, 1.01 | 0.82 | 0.66, 1.01 |  | 0.70 | 0.66, 0.74 | 0.74 | 0.70, 0.78* |
|  | **Number of contracts** | | |  |  |  |  |  |  |  |  |  |
|  | | Continuous | | 0.98 | 0.91, 1.05 | 0.96 | 0.89, 1.03 |  | 1.06 | 1.04, 1.09 | 1.05 | 1.02, 1.07 |
|  | | Discrete categories | |  |  |  |  |  |  |  |  |  |
|  | | 1 | | 1.00 |  | 1.00 |  |  | 1.00 |  | 1.00 |  |
|  | | 2-4 | | 0.96 | 0.78, 1.19 | 0.88 | 0.70, 1.09 |  | 1.07 | 1.00, 1.14 | 1.01 | 0.95, 1.08 |
|  | | >4 | | 0.93 | 0.76, 1.15 | 0.84 | 0.67, 1.04 |  | 1.10 | 1.03, 1.17 | 1.05 | 0.99, 1.12 |
| **Unemployment** | | | |  |  |  |  |  |  |  |  |  |
|  | **Months** | | |  |  |  |  |  |  |  |  |  |
|  | | Continuous | | 0.98 | 0.97, 1.00* | 0.99 | 0.97, 1.00 |  | 1.01 | 1.00, 1.01* | 1.02 | 1.01, 1.02* |
|  | | Discrete categories | |  |  |  |  |  |  |  |  |  |
|  | | 0 | | 1.00 |  | 1.00 |  |  | 1.00 |  | 1.00 |  |
|  | | 1-12 | | 0.84 | 0.66, 1.08 | 0.86 | 0.66, 1.11 |  | 0.93 | 0.86, 1.00 | 0.96 | 0.89, 1.04 |
|  | | >12 | | 0.80 | 0.68, 0.95 | 0.88 | 0.74, 1.05 |  | 1.08 | 1.03, 1.14* | 1.19 | 1.13, 1.25 |
|  | **Number of spells** | | |  |  |  |  |  |  |  |  |  |
|  | | Continuous | | 0.98 | 0.96, 1.00* | 0.99 | 0.97, 1.01 |  | 1.01 | 1.00, 1.01 | 1.02 | 1.01, 1.02* |
|  | | Discrete categories | |  |  |  |  |  |  |  |  |  |
|  | | 0 | | 1.00 |  | 1.00 |  |  | 1.00 |  | 1.00 |  |
|  | | 1 | | 0.68 | 0.55, 0.86* | 0.73 | 0.58, 0.92 |  | 0.92 | 0.86, 0.98* | 1.03 | 0.96, 1.10 |
|  | | >1 | | 0.87 | 0.73, 1.04 | 0.94 | 0.79, 1.13 |  | 1.10 | 1.05, 1.16* | 1.18 | 1.11, 1.24* |
| **Inactivity** | | | |  |  |  |  |  |  |  |  |  |
|  | **Months** | | |  |  |  |  |  |  |  |  |  |
|  | | Continuous | | 1.01 | 0.99, 1.02 | 1.00 | 0.99, 1.02 |  | 1.02 | 1.02, 1.03* | 1.07 | 1.04, 1.09* |
|  | | Discrete categories | |  |  |  |  |  |  |  |  |  |
|  | | 0 | | 1.00 |  | 1.00 |  |  | 1.00 |  | 1.00 |  |
|  | | 1-5 | | 0.99 | 0.79, 1.26 | 1.08 | 0.85, 1.38 |  | 1.00 | 0.94, 1.06 | 1.00 | 0.94, 1.06 |
|  | | >6 | | 1.08 | 0.90, 1.29 | 1.04 | 0.87, 1.25 |  | 1.32 | 1.26, 1.40* | 1.23 | 1.17, 1.30* |
|  | **Number of spells** | | |  |  |  |  |  |  |  |  |  |
|  | | Continuous | | 1.01 | 0.99, 1.03 | 1.01 | 0.99, 1.03 |  | 1.03 | 1.03, 1.04* | 1.02 | 1.02, 1.03* |
|  | | Discrete categories | |  |  |  |  |  |  |  |  |  |
|  | | 0 | | 1.00 |  | 1.00 |  |  | 1.00 |  | 1.00 |  |
|  | | 1 | | 1.17 | 0.94, 1.45 | 1.17 | 0.93, 1.46 |  | 1.08 | 1.02, 1.15* | 1.03 | 0.97, 1.10 |
|  | | >1 | | 1.05 | 0.88, 1.25 | 1.03 | 0.86, 1.24 |  | 1.36 | 1.29, 1.43* | 1.28 | 1.22, 1.35* |
