# Supplementary material for: Employment history indicators and mortality in a nested case-control study from the Spanish WORKing life social security (WORKss) cohort
Source: PLoS One. 2017 Jun 1;12(6):e0178486. doi: 10.1371/journal.pone.0178486 (PMC5453531; doi:10.1371/journal.pone.0178486)
Supplement: S5 Table — a Unadjusted odd ratios. bConfidence Interval. c Adjusted for permanent disability and occupational category. * p-value <0.05 (DOCX) [file pone.0178486.s005.docx]

|  | | |  | **Women** | | | |  | **Men** | | | |
| --- | --- | --- | --- | --- | --- | --- | --- | --- | --- | --- | --- | --- |
|  | | |  | **OR^a^** | **95% CI^b^** | **OR^c^** | **95% CI^b^** |  | **OR^a^** | **95% CI^b^** | **OR^c^** | **95% CI^b^** |
| **Employment** | | | |  |  |  |  |  |  |  |  |  |
|  | **Months** | | |  |  |  |  |  |  |  |  |  |
|  | | Continuous | | 0.99 | 0.94, 1.04 | 0.97 | 0.92, 1.02 |  | 0.85 | 0.81, 0.89* | 0.88 | 0.84, 0.92* |
|  | | Discrete categories | |  |  |  |  |  |  |  |  |  |
|  | | 0-121 (0-9 years) | | 1.00 |  | 1.00 |  |  | 1.00 |  | 1.00 |  |
|  | | 122-197 (10-16 years) | | 1.02 | 0.87, 1.20 | 1.01 | 0.85, 1.19 |  | 0.86 | 0.78, 0.94* | 0.89 | 0.81, 0.98 |
|  | | >197 (>16 years) | | 0.95 | 0.82, 1.11 | 0.95 | 0.81, 1.12 |  | 0.71 | 0.65, 0.77* | 0.75 | 0.69, 0.82* |
|  | **Number of contracts** | | |  |  |  |  |  |  |  |  |  |
|  | | Continuous | | 0.97 | 0.91, 1.04 | 0.93 | 0.87, 1.00* |  | 0.95 | 0.92, 0.99* | 0.93 | 0.89, 0.96* |
|  | | Discrete categories | |  |  |  |  |  |  |  |  |  |
|  | | 1 | | 1.00 |  | 1.00 |  |  | 1.00 |  | 1.00 |  |
|  | | 2-4 | | 1.06 | 0.90, 1.26 | 0.94 | 0.78, 1.12 |  | 0.89 | 0.82, 0.97* | 0.82 | 0.75, 0.90* |
|  | | >4 | | 0.96 | 0.80, 1.14 | 0.85 | 0.70, 1.01 |  | 0.88 | 0.80, 0.96* | 0.81 | 0.74, 0.89* |
| **Unemployment** | | | |  |  |  |  |  |  |  |  |  |
|  | **Months** | | |  |  |  |  |  |  |  |  |  |
|  | | Continuous | | 0.99 | 0.98, 1.01 | 1.00 | 0.99, 1.01 |  | 1.00 | 0.99, 1.01 | 1.00 | 1.00, 1.01 |
|  | | Discrete categories | |  |  |  |  |  |  |  |  |  |
|  | | 0 | | 1.00 |  | 1.00 |  |  | 1.00 |  | 1.00 |  |
|  | | 1-12 | | 0.79 | 0.65, 0.96* | 0.85 | 0.69, 1.04 |  | 0.92 | 0.82, 1.02 | 0.90 | 0.80, 1.00 |
|  | | >12 | | 1.00 | 0.87, 1.15 | 1.02 | 0.88, 1.18 |  | 1.02 | 0.94, 1.09 | 1.06 | 0.98, 1.14 |
|  | **Number of spells** | | |  |  |  |  |  |  |  |  |  |
|  | | Continuous | | 0.99 | 0.98, 1.01 | 0.99 | 0.98, 1.01 |  | 1.00 | 0.99, 1.01 | 1.00 | 0.99, 1.01 |
|  | | Discrete categories | |  |  |  |  |  |  |  |  |  |
|  | | 0 | | 1.00 |  | 1.00 |  |  | 1.00 |  | 1.00 |  |
|  | | 1 | | 0.86 | 0.72, 1.03 | 0.91 | 0.75, 1.10 |  | 0.95 | 0.87, 1.04 | 1.00 | 0.91, 1.09 |
|  | | >1 | | 0.96 | 0.84, 1.11 | 0.98 | 0.85, 1.14 |  | 0.98 | 0.91, 1.07 | 1.00 | 0.92, 1.08 |
| **Inactivity** | | | |  |  |  |  |  |  |  |  |  |
|  | **Months** | | |  |  |  |  |  |  |  |  |  |
|  | | Continuous | | 1.00 | 0.99, 1.00 | 0.99 | 0.98, 1.00 |  | 1.01 | 1.00, 1.01* | 1.00 | 1.00, 1.01 |
|  | | Discrete categories | |  |  |  |  |  |  |  |  |  |
|  | | 0 | | 1.00 |  | 1.00 |  |  | 1.00 |  | 1.00 |  |
|  | | 1-5 | | 1.01 | 0.83, 1.24 | 0.97 | 0.79, 1.19 |  | 0.95 | 0.87, 1.05 | 0.92 | 0.83, 1.01 |
|  | | >6 | | 0.96 | 0.83, 1.09 | 0.88 | 0.76, 1.01 |  | 1.12 | 1.02, 1.20* | 1.02 | 0.94, 1.10 |
|  | **Number of spells** | | |  |  |  |  |  |  |  |  |  |
|  | | Continuous | | 1.00 | 0.98, 1.01 | 0.99 | 0.97, 1.00 |  | 1.01 | 1.00, 1.02 | 1.00 | 0.99, 1.01 |
|  | | Discrete categories | |  |  |  |  |  |  |  |  |  |
|  | | 0 | | 1.00 |  | 1.00 |  |  | 1.00 |  | 1.00 |  |
|  | | 1 | | 1.00 | 0.84, 1.19 | 0.94 | 0.78, 1.13 |  | 1.02 | 0.93, 1.12 | 0.95 | 0.86, 1.04 |
|  | | >1 | | 0.97 | 0.84, 1.11 | 0.89 | 0.77, 1.04 |  | 1.16 | 1.06, 1.26* | 1.07 | 0.98, 1.17 |
