# Supplementary material for: Employment history indicators and mortality in a nested case-control study from the Spanish WORKing life social security (WORKss) cohort
Source: PLoS One. 2017 Jun 1;12(6):e0178486. doi: 10.1371/journal.pone.0178486 (PMC5453531; doi:10.1371/journal.pone.0178486)
Supplement: S7 Table — a Unadjusted odd ratios. bConfidence Interval. c Adjusted for permanent disability and occupational category. * p-value <0.05 (DOCX) [file pone.0178486.s007.docx]

|  | | |  | **Women** | | | |  | **Men** | | | |
| --- | --- | --- | --- | --- | --- | --- | --- | --- | --- | --- | --- | --- |
|  | | |  | **OR^a^** | **95% CI^b^** | **OR^c^** | **95% CI^b^** |  | **OR^a^** | **95% CI^b^** | **OR^c^** | **95% CI^b^** |
| **Employment** | | | |  |  |  |  |  |  |  |  |  |
|  | **Months** | | |  |  |  |  |  |  |  |  |  |
|  | | Continuous | | 0.86 | 0.78, 0.95* | 0.84 | 0.75, 0.93* |  | 0.45 | 0.42, 0.49* | 0.54 | 0.50, 0.58* |
|  | | Discrete categories | |  |  |  |  |  |  |  |  |  |
|  | | 0-121 (0-9 years) | | 1.00 |  | 1.00 |  |  | 1.00 |  | 1.00 |  |
|  | | 122-197 (10-16 years) | | 0.87 | 0.71, 1.07 | 0.77 | 0.63, 0.97* |  | 0.64 | 0.57, 0.74* | 0.64 | 0.56, 0.73* |
|  | | >197 (>16 years) | | 0.78 | 0.66, 0.93* | 0.75 | 0.62, 0.90* |  | 0.40 | 0.36, 0.45* | 0.46 | 0.41, 0.52* |
|  | **Number of contracts** | | |  |  |  |  |  |  |  |  |  |
|  | | Continuous | | 1.01 | 0.96, 1.07 | 0.94 | 0.88, 1.01 |  | 1.14 | 1.12, 1.17* | 1.07 | 1.04, 1.10* |
|  | | Discrete categories | |  |  |  |  |  |  |  |  |  |
|  | | 1 | | 1.00 |  | 1.00 |  |  | 1.00 |  | 1.00 |  |
|  | | 2-4 | | 1.03 | 0.86, 1.22 | 0.91 | 0.74, 1.11 |  | 1.01 | 0.94, 1.09 | 0.94 | 0.86, 1.02 |
|  | | >4 | | 1.06 | 0.89, 1.25 | 0.82 | 0.67, 1.01 |  | 1.23 | 1.15, 1.32* | 1.02 | 0.94, 1.11 |
| **Unemployment** | | | |  |  |  |  |  |  |  |  |  |
|  | **Months** | | |  |  |  |  |  |  |  |  |  |
|  | | Continuous | | 1.01 | 1.00, 1.02 | 1.00 | 0.99, 1.02 |  | 1.03 | 1.02, 1.03* | 1.02 | 1.01, 1.02* |
|  | | Discrete categories | |  |  |  |  |  |  |  |  |  |
|  | | 0 | | 1.00 |  | 1.00 |  |  | 1.00 |  | 1.00 |  |
|  | | 1-12 | | 0.84 | 0.69, 1.01 | 0.83 | 0.67, 1.03 |  | 1.03 | 0.96, 1.10 | 0.97 | 0.89, 1.04 |
|  | | >12 | | 1.12 | 1.00, 1.27 | 1.06 | 0.92, 1.23 |  | 1.37 | 1.30, 1.44* | 1.22 | 1.16, 1.29* |
|  | **Number of spells** | | |  |  |  |  |  |  |  |  |  |
|  | | Continuous | | 1.01 | 0.99, 1.02 | 1.00 | 0.99, 1.02 |  | 1.03 | 1.03, 1.04* | 1.02 | 1.01, 1.02* |
|  | | Discrete categories | |  |  |  |  |  |  |  |  |  |
|  | | 0 | | 1.00 |  | 1.00 |  |  | 1.00 |  | 1.00 |  |
|  | | 1 | | 0.87 | 0.72, 1.04 | 0.83 | 0.68, 1.02 |  | 1.03 | 0.96, 1.10 | 1.01 | 0.94, 1.09 |
|  | | >1 | | 1.12 | 0.99, 1.26 | 1.06 | 0.92, 1.23 |  | 1.35 | 1.29, 1.42* | 1.18 | 1.12, 1.25* |
| **Inactivity** | | | |  |  |  |  |  |  |  |  |  |
|  | **Months** | | |  |  |  |  |  |  |  |  |  |
|  | | Continuous | | 1.01 | 1.00, 1.02 | 1.00 | 0.99, 1.01 |  | 1.04 | 1.03, 1.04* | 1.02 | 1.02, 1.03* |
|  | | Discrete categories | |  |  |  |  |  |  |  |  |  |
|  | | 0 | | 1.00 |  | 1.00 |  |  | 1.00 |  | 1.00 |  |
|  | | 1-5 | | 1.15 | 0.94, 1.39 | 1.06 | 0.86, 1.30 |  | 1.10 | 1.03, 1.18* | 1.00 | 0.93, 1.07 |
|  | | >6 | | 1.12 | 0.99, 1.27 | 0.98 | 0.85, 1.12 |  | 1.51 | 1.43, 1.58* | 1.24 | 1.17, 1.31* |
|  | **Number of spells** | | |  |  |  |  |  |  |  |  |  |
|  | | Continuous | | 1.02 | 1.00, 1.03* | 1.00 | 0.98, 1.02 |  | 1.05 | 1.04, 1.05* | 1.03 | 1.02, 1.03* |
|  | | Discrete categories | |  |  |  |  |  |  |  |  |  |
|  | | 0 | | 1.00 |  | 1.00 |  |  | 1.00 |  | 1.00 |  |
|  | | 1 | | 1.18 | 1.01, 1.39* | 1.10 | 0.92, 1.31 |  | 1.14 | 1.07, 1.22* | 1.04 | 0.97, 1.12 |
|  | | >1 | | 1.13 | 1.00, 1.28* | 0.97 | 0.84, 1.12 |  | 1.58 | 1.50, 1.66* | 1.30 | 1.23, 1.38* |
